# Nephrotoxicity of Chloroquine and Hydroxychloroquine in COVID-19 Patients

**DOI:** 10.34172/apb.2021.022

**Published:** 2020-11-07

**Authors:** Javad Mahmoudi, Saeed Sadigh-Eteghad, Hanieh Salehi-Pourmehr, Afshin Gharekhani, Mojtaba Ziaee

**Affiliations:** ^1^Neurosciences Research Center, Tabriz University of Medical Sciences, Tabriz, Iran.; ^2^Research Center for Evidence-Based Medicine, Faculty of Medicine, Tabriz University of Medical Sciences, Tabriz, Iran.; ^3^Department of Clinical Pharmacy, Faculty of Pharmacy, Tabriz University of Medical Sciences, Tabriz, Iran.; ^4^Medicinal Plants Research Center, Maragheh University of Medical Sciences, Maragheh, Iran.

## Dear Editor,

In November 2002, severe acute respiratory syndrome coronavirus (SARS-CoV) emerged in china, and several months later caused over 8000 cases in 26 countries. 17 years later in December 2019, a novel coronavirus, known as SARS-CoV-2, which causes COVID-19 disease in humans reported in Wuhan China and considered global pandemic as its high contagious properties. In January 2020, World Health Organization (WHO) declared COVID-19 as an international public health emergency. As of June 2020, COVID-19 is responsible for more than 6 million confirmed cases and more than 377 000 deaths in 215 countries. There are many interesting similarities between SARS-CoV and SARS-CoV-2, not only in name.^1-3^ COVID-19 patients in most cases experience mild symptoms such as dry cough, fever, chills, and sore throat with spontaneous healing, but sometime symptoms may worsen and could develop life-threatening conditions such as acute respiratory distress syndrome, septic shock, pulmonary edema, organ failure and severe pneumonia.^4^ Current situation of disease and spreading pattern shows that health care professionals are suspected of being highly morbid both because of close contact in hospital conditions with infected patients as well as evidence of asymptomatic transmission of the disease. Regrettably, no pharmaceutical products (vaccine or medicine) have fully approved by regulatory authorities for the prevention or treatment of COVID-19 disease so far. By June 2020, the U.S. Food and Drug Administration (FDA) issued emergency authorization for the investigational remdesivir in COVID-19 hospitalized patients.^5^

In light of the inadequate impact that alternative non-pharmacological strategies on disease mitigation various available antiviral therapies are being tested against this emerging disease yet. For decades, Chloroquine/ Hydroxychloroquine (CQ/HCQ) have been used as anti-malaria medications and also for their immunomodulatory effects in conditions including rheumatoid arthritis disease and systemic lupus erythematosus.^6^

CQ and its β-hydroxylated analog, HCQ have a large distribution volume and demonstrate similar pharmacokinetic patterns in body organs. CQ is rapidly absorbed in the gastrointestinal tract. The CQ has an approximate half-life of 50 days and a high renal clearance, making the bioavailability of CQ an important clinical factor for kidney failure patients.^7^ While HCQ has a half-life of 30 days and takes about 6 months to fully eliminate the body. These medications are excreted in the milk.^8^ The safety margin of chloroquine is low. Recent experiments reported that CQ/ HCQ efficiently inhibited COVID-19 infection.^9,10^ Some guidelines suggested prophylactic strategies for those accidentally exposed to SARS-CoV-2 to prevent viral transmission. CQ and HCQ may interfere with virus replication at the early stages of infection or increasing endosome-mediated pH at the viral entry/cell fusion, as well as post-translational modification of the glycosylation of cellular receptors of SARS-CoV-2.^6^ Along with this, Savarino et al suggested that CQ may reduce the level of inflammatory cytokines and could ameliorate acute respiratory distress syndrome.^11^ Recent preliminary clinical studies suggested that CQ and HCQ may be able to lower the COVID-19 viral load and reduce the duration of viremia.

Drug-induced renal failure is a common pharmacological reaction, while the precise mechanism of drug-induced renal failure remains unclear yet. However, some reports suggest that between 5 and 20% of cases of acute renal failure can be directly attributed to drugs and chemicals, although minor damage may pass undetected. It is important to be aware of the types of drugs that can induce renal impairment because, if suspected and acted on early, the damage to the kidney may be reversible. The renal system provides the ultimate solution for the excretion and eliminations of many xenobiotics and metabolites, often subject to high levels of potentially toxic substances. As a result, the renal tubular cell and the renal papillae typically experience direct toxic damages. This form of nephrotoxicity is typically dose-dependent. Many groups of medicines that cause renal injuries, and in patients who have pre-existing renal injury these effects are distinct. Wang et al showed that chronic CQ consumption has a devastating effect on glomerulus as well as acute distal tubular cell apoptosis in rats.^12^ Previous studies showed that SARS-CoV-2 binds to an enzyme called angiotensin-converting enzyme 2 (ACE_2_) receptor on the surface of human tissues.^13,14^ ACE_2_ Receptors distributed in body organs such as lung, heart, and renal tubular cells and upregulated by COVID-19 infection.^14^ Therefore, the virus could specifically invade positive ACE_2_ cells and lesion target tissues. After respiratory infection, the virus infiltrates the blood circulation and could persist in the kidney and trigger renal tissue injury. The rate of kidney dysfunction in hospitalized COVID-19 patients is high.^13,15,16^ This devastating synergism between SARS-CoV-2 and CQ/HCQ could be a lethal combination for patients in critical care units and would affect patients even after discharge from the hospital. Based on these investigations, we believe that the renal damage should be considered in COVID-19 patients during clinical work, because of the kidney impairment caused by both viral infection and CQ/HCQ treatment with certain renal toxicity.^12^ Physicians should increase their attention to renal failure in aged COVID-19 patients who received CQ/HCQ to reduce deaths. Furthermore, due to the potential pathogenicity of the virus to the kidney as well as the respiratory system, clinicians should pay attention to the risk of renal lesions in patients during the bedridden period and later clinical follow-up.

## Ethical Issues

Not applicable.

## Conflict of Interest

Authors of this paper declare no financial competing interests.
